# The risk factors of failed reimplantation arthroplasty for periprosthetic hip infection

**DOI:** 10.1186/s12891-017-1622-1

**Published:** 2017-06-12

**Authors:** Shun-Wun Jhan, Yu-Der Lu, Mel S. Lee, Chen-Hsiang Lee, Jun-Wen Wang, Feng-Chih Kuo

**Affiliations:** 1 Department of Orthopaedic Surgery, Kaohsiung Chang Gung Memorial Hospital, No. 123, Ta Pei Road, Niao Sung Dist, Kaohsiung City, 833 Taiwan; 2 Division of Infectious Diseases, Department of Internal Medicine, Kaohsiung Chang Gung Memorial Hospital, Kaohsiung, Taiwan; 3 Chang Gung University College of Medicine, Kaohsiung, Taiwan

**Keywords:** Reimplantation, Hip arthroplasty, Periprosthetic joint infection, Risk factor

## Abstract

**Background:**

Two-stage reimplantation arthroplasty is one of the standard treatments for chronic periprosthetic joint infection (PJI). Scanty data exist regarding the risk factors for failure after two-stage reimplantation for periprosthetic hip infection. The purpose of this study was to investigate and identify the risk factors associated with failure after two-stage reimplantation hip arthroplasty.

**Methods:**

Sixty-two patients with hip PJI treated with a two-stage reimplantation protocol at our institution from 2005 to 2012 were reviewed. Patients requiring medical treatment or reoperation for recurrent infection were defined as treatment failure. A multivariate Cox proportional hazards model was used to analyze the risk factors associated with treatment failure.

**Results:**

Of the 62 patients, 11 (17.7%) patients had developed reinfection after the two-stage reimplantation with a mean follow-up of 5.7 years. The implant survival was 82.2% (95% confidence interval [CI] 75.19−92.55) at 10 years. Multivariate analysis revealed BMI ≥30 kg/m^2^ (hazard ratio [HR] 9.16; 95% CI 1.51−55.3; *p* = 0.0158), liver cirrhosis (HR 6.39; 95% CI 1.09−37.4; *p* = 0.0398), gram-negative organism (HR 5.68; 95% CI 1.18−27.4; *p* = 0.0303), and presence of sinus tract (HR 18.2; 95% CI 2.15−153; *p* = 0.0077) as the independent risk factors for treatment failure.

**Conclusions:**

We found obesity, liver cirrhosis, gram-negative organism, and the presence of sinus tract were significantly related to the risks of failure after reimplantation arthroplasties.

## Background

Infection is the third leading cause for revision after total hip arthroplasty (THA) [1] and the most common cause of failure after revision THA [2]. The incidence of periprosthetic joint infection (PJI) following THA varied from 0.5 to 2.2% [3]. Hip PJI also result in huge economic burden and medical resource utilization in the United States and other countries [4].

The two-stage reimplantation arthroplasty is one of the standard treatments for chronic infected hip prosthesis in the United States and many countries [5]. The goals of successful treatment included eradication of infection and restoration of function. Usually it can reach more than 90% successful rate of infection eradication [6]. However, recurrence and repetitive infections may cause troublesome complications such as bone loss, poor soft tissue integrity, prolonged and complex operation, and physical and psychological disabilities.

Several risk factors related to PJI in THA and revision THA have been well documented in previous literatures, including age [7], male [8], obesity [9], comorbidities with rheumatoid arthritis [10], diabetes [11] liver cirrhosis [12]. However, few studies have been done to analyze the risk factors of failed reimplantation protocol [6, 13]. The purpose of this study was to investigate the patient-related risk factors associated with treatment failure following the reimplantation protocol in hip PJI.

## Methods

After Institutional Review Board approval, we retrospectively reviewed patients who underwent two-staged reimplantation arthroplasties for periprosthetic joint infection (PJI) of hips between January 2005 and December 2012. Eligible patients with a minimum follow-up for 2 years were included. The exclusion criteria were patients with incomplete medical data, unconfirmed diagnosis of PJI, or less than 2 years follow-up.

PJI was confirmed according to the Musculoskeletal Infection Society (MSIS) guidelines [14] with one major criteria or three out of five minor criteria. All procedures were performed through a posterolateral approach. At the first stage, it included excision of sinus tracts, removal of prosthesis, radical surgical debridement, and implantation of antibiotic-loaded cement beads. The regimen of antibiotics in the bone cement was determined according the culture results from preoperative joint aspiration or previous culture report. If the infecting microorganism could not be known at the time of resection arthroplasty, empirical combination of 2–4 g vancomycin and 2–4 g piperacillin per 40 g package of bone cement was used. During surgery, at least three sets of tissue specimens were sent for culture. Further debridement or exchange to sensitive antibiotic-loaded cement beads may be needed if the infection could not be controlled during the interim stage. According to an infectious disease specialist suggestion, culture-specific parenteral antibiotics were given postoperatively for 4 weeks, followed by oral antibiotics for 2 weeks. The timing of the second stage reimplantation was based on clinical condition and laboratory data. Reimplantation was performed after an at least 2-week antibiotic holiday without elevation of erythrocyte sedimentation rate (ESR) and C-reactive protein (CRP). In patients with other underlying diseases such as gout, autoimmune disease or other chronic diseases, the ESR and serum CRP level may not return to normal. In these patients, we performed the second reimplantation according to the clinical condition combined with a trend of decreased ESR and CRP levels after discontinuing oral antibiotics.

We divided our patients into successful and reinfection groups. The successful group was defined as functioning and stable joints without any evidence of reinfection. The reinfection group included patients who had recurrent infection, needed antibiotics suppression, or required reoperation for PJI.

Multiple potential predictive variables were collected from medical record, including patients’ characteristics, comorbidities, causative organism and the operation-related factors. Minimal follow-up was at least 2 years after reimplantation.

### Statistical analysis

Categorical variables are expressed as count and percentage, and continuous data as mean ± standard deviation (SD) and range. Proportional hazard regression univariate and multivariate analysis were performed to assess the association of clinically interesting covariates with the risk of recurrent infection. Hazard ratios and 95% confidence intervals (CIs) for the risk of recurrent infection of the exposure variables were reported. The survival rate free of infection was estimated with the use of Kaplan-Meier survival curve. The survival end point was defined as recurrent infection when repeated operations were necessary after definite reimplantation. Log-rank test was performed for Kaplan-Meier survival analysis between significant variables. *P* values < 0.05 were considered statistically significant. Statistical analysis was completed by using software (version 14.12.0; MedCalc, Ostend, Belgium).

## Results

A total of 62 PJI in 62 patients (43 males and 19 females) were included. The mean age at the time of first stage operation was 57 ± 14 years (range: 27–86 years). The mean BMI was 25.3 ± 4.6 kg/m^2^ (range: 17.7−39.8 kg/m^2^). The mean interim period before reimplantation was 20 ± 15.8 weeks (range: 8–104 weeks). The mean follow-up was 5.7 ± 2.4 years (range: 2–10 years). The overall successful rate was 82.3%. Eleven hips (17.7%) had recurrence of PJI and required subsequent surgeries. Two of them underwent repeated two-stage reimplantation. One patient received debridement-antibiotic-implant retention. Eight patients had permanent resection arthroplasty procedures, including two patients with HIV (human immunodeficiency virus) and continuous use of drug abuse after reimplantation.

Table 1 presented univariate risk factors for treatment failure. Significant risk factors were BMI≥30 kg/m^2^ (HR 4.92; 95% CI 1.50−16.1; *p* = 0.0085), illicit drug abuse (HR 10.1; 95% CI 2.11−48.0; *p* = 0.0037), liver cirrhosis (HR 7.94; 95% CI 2.39−26.2; *p* = 0.0007), gram-negative organism (HR 4.83; 95 CI 1.40−16.6; *p* = 0.0124), the presence of a sinus tract (HR 9.24; 95% CI 2.66−32.0; *p* = 0.0005), repeated debridement between stage (HR 13.7; 95% CI 1.75−107; *p* = 0.0125) and operation time>4 h (HR 8.47; 95% CI 1.08-66.3; *p* = 0.0419). In the multivariate Cox regression analysis, the following factors were independent risks for treatment failure: BMI≥30 kg/m^2^ (HR 9.16; 95% CI 1.51−55.3; *p* = 0.0158), liver cirrhosis (HR 6.39; 95% CI 1.09−37.4; *p* = 0.0398), gram-negative organism (HR 5.68; 95% CI 1.18−27.4; *p* = 0.0303), and presence of sinus tract (HR 18.2; 95% CI 2.15−153; *p* = 0.0077) (Table 2).

**Table 1:** Univariate analysis for risk factors associated with reinfection of two-stage revision THAData are mean (range) or number (%) of episodes

	Variable	Success (*N* = 51)		Reinfection (*N* = 11)		HR (95% CI)	*p* value
	Age (years)	57.8	(27–86)	53.2	(42–79)	0.97 (0.93–1.02)	0.3621
	Male	36	(70%)	7	(63%)	1.44 (0.42–4.93)	0.5587
Patient characteristics	BMI (kg/m^2^)						
	<30	43	(84%)	5	(46%)	–	
	≥30	8	(16%)	6	(54%)	4.92 (1.50–16.1)	0.0085^*^
	Smoking	17	(33%)	5	(45%)	1.55 (0.47–5.11)	0.4641
	ASA						
	2	28	(55%)	3	(27%)	–	
	3	23	(45%)	8	(73%)	2.97 (0.78–11.2)	0.1077
	Diabetes mellitus	8	(16%)	2	(18%)	1.24 (0.26–5.78)	0.7764
	Hepatitis	10	(20%)	4	(36%)	2.16 (0.63–7.40)	0.2176
Medical diseases	Gout	6	(12%)	3	(27%)	2.27 (0.60–8.57)	0.2257
	CKD	5	(10%)	1	(9%)	1.00 (0.12–7.86)	0.9987
	ESRD	1	(2%)	1	(9%)	3.96 (0.50–31.2)	0.1910
	Drug abuse	0	(0%)	2	(18%)	10.1 (2.11–48.0)	0.0037^*^
	Liver cirrhosis	4	(9%)	6	(54%)	7.94 (2.39–26.2)	0.0007^*^
	Autoimmune disease	3	(6%)	1	(9%)	1.81 (0.22–14.4)	0.5579
	*Staphylococcus aureus*	7	(13.7%)	2	(18.1%)	1.43 (0.31–6.67)	0.6425
	*Coagulase-negative Staphylococcus*	1	(1.9%)	1	(9%)	3.46 (0.43–27.7)	0.2413
Microbiology	Methicillin-resistant organism	10	(19.6%)	1	(9%)	0.43 (0.05–3.40)	0.4287
	Gram-negative organism	4	(9%)	4	(36.3)	4.83 (1.40–16.6)	0.0124^*^
	Polymicrobial organism	4	(9%)	1	(9%)	1.57 (0.20–12.3)	0.6651
	Culture-negative	16	(31.3%)	2	(18.1%)	0.49 (0.10–2.28)	0.3671
	Presence of sinus tract	6	(12%)	7	(63%)	9.24 (2.66–32.0)	0.0005^*^
	Interim period less than 3 months	9	(17%)	3	(27%)	1.78 (0.47–6.74)	0.3949
Operation-related factors	Repeated debridement between stage	18	(35%)	10	(91%)	13.7 (1.75–107)	0.0125^*^
	Perioperative Blood loss >1500 ml	18	(35%)	5	(45%)	1.37 (0.41–4.49)	0.6031
	Revision operative time >4 h	26	(51%)	10	(91%)	8.47 (1.08–66.3)	0.0419^*^
	Allograft use in revision	42	(82%)	9	(82%)	1.04 (0.22–4.83)	0.9586

*BMI* body mass index, *ASA* American Society of Anesthesiologist, *ESRD* end stage renal disease, *CKD* chronic kidney disease, *HR* hazard ratio, *CI* confidence interval

*A *p* value of <0.05 was considered to be statistically significant

**Table 2:** Multivariate analysis for risk factors associated with reinfection after two-stage revision THA

Variables	Adjusted HR	95% CI	*p* value
Body mass index ≥30 kg/m^2^	9.16	1.51−55.3	0.0158^*^
Drug abuse	1.66	0.14−19.4	0.6832
Liver cirrhosis	6.39	1.09−37.4	0.0398^*^
Gram-negative organism	5.68	1.18−27.4	0.0303^*^
Presence of sinus tract	18.2	2.15−153	0.0077^*^
Repeated debridement between stage	5.64	0.59−53.1	0.1303
Revision operative time >4 h	1.00	0.08−12.5	0.9957

*HR* hazard ratio, *CI* confidence interval

*A *p* value of <0.05 was considered to be statistically significant

Among the reinfection group, one patient was considered a relapse case because of the same pathogenic organism with initial microbiology. One patient still had had a negative culture result at the interim stage and the reinfection period. The other 9 patients in the reinfection group had different culture results from initial microorganisms (Table 3).

**Table 3:** Microbiology of the reinfection group

Case	Initial	Reinfection
1	*Coagulase-negative Staphylococcus*	MSSA
2	Culture-negative	*Viridans streptococcus*
3	Polymicrobial	MSSA
4	MSSA	*Klebsiella pneumonia*
5	MRSA	Polymicrobial
6	Culture-negative	Culture-negative
7	*Enterobacter Cloacae*	MRSA
8	*E. coli*	*Pseudomonas aeruginosa*
9	MSSA	*Pseudomonas aeruginosa*
10	*E. coli*	*E. coli*
11	ESBL-producing *E. coli*	*E. coli*

*MSSA* Methicillin-sensitive *Staphylococcus aureus*, *MRSA* Methicillin-resistant Staphylococcus aureus, *E. coli Escherichia coli*, *ESBL* extended-spectrum β-lactamase

Table 4 showed the drug susceptibilities of the gram-negative microorganisms. Among the success group, two patients had *Escherichia coli* infection. One was treated with cefazolin and the other was treated with cefuroxime. In the patient with *Pseudomonas aeruginosa* infection, piperacillin was administrated during hospitalization and oral ciprofloxacin was prescribed after discharge. The patient with *Klebsiella pneumoniae* infection was treated with piperacillin/tazobactam. Among the reinfection group, one patient with *Escherichia coli* infection was treated with cefazolin. Two patients with *Enterobacter Cloacae* and *E. coli* infection showed resistance to cefazolin and were treated with ceftazidime. In a patient with extended-spectrum beta-lactamase-producing *Escherichia coli* infection, imipenem was administrated.

**Table 4:** The drug susceptibilities of gram-negative microorganisms

	Cefazolin	Cefuroxime	Ceftazidime	Ciprofloxacin	Gentamicin	Piperacillin	Piperacillin/tazobactam	Imipenem	Sulfamethoxazole-Trimethoprim
Success group									
*E. coli*	S^a^	S	S	S	S	S	S	S	S
*E. coli*	I	S^a^	S	S	R	R	S	S	R
*Pseudomonas aeruginosa*	R	R	S	S^a^	S	S^a^	S^a^	S	R
*Klebsiella pneumoniae*	S	S	S	S	S	S	S	S	S
Reinfection group									
*Enterobacter Cloacae*	R	R	S^a^	S	S	S	S	S	R
*E. coli*	R	S	S^a^	S	S	R	S	S	S
*E. coli*	S^a^	S	S	S	S	R	S	S	S
*E. coli – ESBL*	R	R	R	R	R	R	S	S^a^	R

*E. coli Escherichia coli, ESBL extended-spectrum β-lactamase*

^a^means the systemic antibiotic treatment

**Fig. 1:**
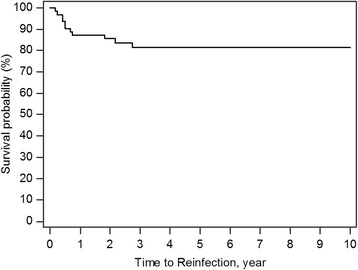
Kaplan-Meier survival for implant from two-stage revision total hip arthroplasty to reinfection. The 10-year survivorship with infection-free was 82.2% (95% CI 75.19–92.55)

**Fig. 2:**
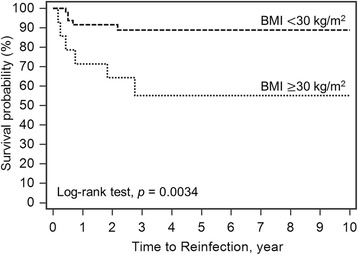
Log-rank test for Kaplan-Meier survival analysis in BMI. The 10-year survivorship with infection-free was 55.1% in the patients with BMI ≥30 kg/m^2^ versus 88.9% in those with BMI <30 kg/m^2^

**Fig. 3:**
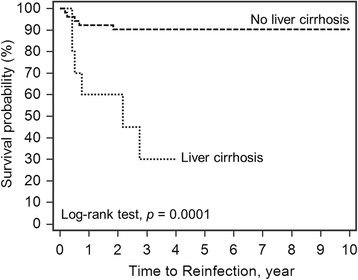
Log-rank test for Kaplan-Meier survival analysis in liver cirrhosis. The 10-year survivorship with infection-free was 90.4% in patients without liver cirrhosis and 30% in liver cirrhotic patients

**Fig. 4:**
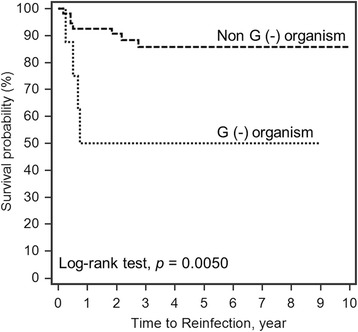
Log-rank test for Kaplan-Meier survival analysis in PJI caused by Gram-negative organisms. The 10-year survivorship with infection-free was 85.8% in patients without gram negative PJI and 50% in patients with gram negative PJI

**Fig. 5:**
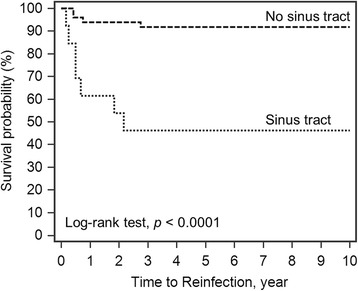
Log-rank test for Kaplan-Meier survival analysis in presence of sinus tract. The 10-year survivorship with infection-free was 43.1 and 91.2% in patients with and without sinus tract

The 10-year survivorship of the implant with infection-free after two-stage reimplantation was 82.2% (95% CI 75.19−92.55) (Fig. 1). The Log-rank test for Kaplan-Meier survival analysis for risk factor of treatment failure showed a significantly lower survival rate in obese patients (55.1%; *p* = 0.0034, Fig. 2), liver cirrhosis (30%; *p* = 0.0001, Fig. 3), gram-negative bacillus (50%; *p* = 0.0050, Fig. 4) and presence of sinus tract (43.1%; *p* < 0.0001, Fig. 5).

## Discussion

In this representative cohort of 62 patients who underwent two-stage reimplantation arthroplasties for PJI, the 10-year survival rate for implant with infection-free was 82.2 % (95% CI 75.19−92.55). After considering risk factors for reinfection, we found patients with BMI≥30 kg/m^2^, liver cirrhosis, gram-negative bacteria and the presence of sinus tract had significantly higher risks for treatment failure.

The infection eradication of two-stage exchange arthroplasty has been reported to be around 90 % in other literatures. Sanchez-Sotelo et al. reviewed 168 patients with infected THA who underwent two-staged revision THA and found the rate of reinfection was 7.1% at a mean follow-up of 7 years [15]. Haddad et al. followed up 50 patients treated according to a two-staged protocol and found the rate of reinfection was 8% at a mean follow-up of 5.8 years [16]. Chen et al. retrospectively reviewed 155 patients (157 hips) who received two-staged revision THA for hip PJI and the infection-free successful rate was 91.7% at an average of 9.7 years [6]. In our study, the reinfection rate was 17.7% at a mean follow-up of 5.7 years, which was relative high than previous studies.

Obesity had been associated with PJI following THA and revision THA [7, 17]. Spiegl et al. reported the risk factors for failed two-stage procedure after chronic hip PJI in 26 patients. They found a high BMI was one of risk factors for treatment failure [18]. In another matched-control study compared with non-obese patients (BMI<30 kg/m^2^), morbidly obese patients (BMI≥40 kg/m^2^) had increased rate of reinfection following revision THA for periprosthetic joint infection [19]. In these studies, they used univariate variables for risk factor analysis. It may be interfered by other confounding factors. Tikhilov et al. used total risk score to calculate the risk of infection recurrence after two-stage procedure. They found BMI was identified as one of main factors related to infection recurrence [13]. Our study agreed with Tikhilov et al. We used multivariate analysis and found BMI≥30 kg/m^2^ was associated with an increased risk for treatment failure following two-stage reimplantation.

Liver cirrhosis was another significant risk factor of treatment failure in this study. In a retrospective study of 38 cirrhotic patients after hip arthroplasties, liver cirrhosis had been associated with PJI, which was the most common cause of treatment failure [20]. In another cohort of 20 cirrhotic patients with PJI undergoing two-stage reimplantation, a higher risk of recurrent infection was noted in these cirrhotic patients with decompensated liver function [12] Chen et al. also mentioned liver cirrhosis was an independent risk factor of recurrent infection [6]. Patients with liver cirrhosis had decreased ability to activate reticuloendothelial system, neutrophil mobilization and phagocytic activity [21]. Thus, their bactericidal activity is diminished. The immune compromised status of liver cirrhosis patients should be more stringently monitored before reimplantation to decrease chance of treatment failure.

Little literature exists with regard to the outcome of PJI caused by gram-negative organisms. Hsieh et al. reported 16 patients with GN-PJI underwent two-stage exchanged arthroplasty. The 2-year survival rate free of treatment failure for patients with GN PJI was 87% (95% CI 80–99) for two-stage exchange. They also found a similar outcome to those patients treated for gram-positive PJI [22]. Zmistowski et al. reported a 52% overall success in treating GN-PJI, which was consistent with our findings [23]. We had 8 GN-PJI with a 50% failure rate by the two-stage reimplantation protocol. In the success group, two patients had *Escherichia coli*, one had *Pseudomonas aeruginosa* and one had *Klebsiella pneumoniae*. In the reinfection group, two patients had *Escherichia coli*, one had *Enterobacter cloacae* and one had extended-spectrum β-lactamases (ESBL)-producing *Escherichia coli*. Patients with GN organism PJI had 5.8 times of treatment failure as compared with other organisms. We thought the low successful rate might be related to antibiotics in the bone cement. In our study, we used 2–4 g piperacillin in a 40−g bone cement. But three patients in the reinfection group had the drug susceptibilities showing resistance to piperacillin. Piperacillin-loaded bone cement was unable to provide antibacterial duration more than 24 h against the gram-positive and gram-negative microorganisms in vitro study [24]. Therefore, we have used ceftazidime instead of piperacillin for better local antibiotic release from the bone cement for the treatment of gram-negative PJI in our institute since January 2015. Further studies should be conducted to compare the ceftazidime and piperacillin as the local antibiotic regimen in the treatment of periprosthetic hip infection.

Presence of sinus tract as an independent risk factor for treatment failure in two-stage revision is scanty reported. Betsch et al. found presence of sinus tract was a risk factor for treatment failure in univariate analysis (HR 2.35; 95 % CI 1.10−5.0; *p* = 0.02) but not an independent risk factor in multivariate analysis [25]. In our study, we found presence of sinus tract was an independent risk factor for treatment failure (HR 18.2; 95% CI 2.15−153; *p* = 0.0077) after multivariate analysis and had lower 10-year survival rate than patients without sinus tract. In this series, sinus tracts and the superficial contaminated layers were excised at the first-stage surgery and all the surgical wounds could be closed by primary suture. No further plastic surgery was involved to perform soft tissue coverage and dead space management. The discharge sinus and fistula tract are often combined with abscess cavity and scarred tissue that are difficult to heal and collapse. If the dead space is not treated by plastic surgeons, patients with liver disease or gram negative rods infection will have wound healing problems and haematoma after surgery. It is becoming evident that adequate soft tissue coverage, usually with muscle flaps to occult the dead space after radical debridement, is paramount to the success of two stage revisions for periprosthetic joint infection [26]. The suboptimal management of dead space could explain the high percentage of repeated debridement (up to 45%) and an increased reinfection rate in relation to the average in our patients.

There were limitations of our study. First, this was a retrospective study. Second, our cohort is small and is derived from a tertiary referral medical center. The complexity of our cases may be higher than other reports. Third, the mean follow-up was 5.7 years (range: 2–10 years) that the reinfection rate might be accumulated with longer term of follow up. Fourth, the choice and the duration of parenteral and local antibiotics were not standardized. Another limitation to keep in mind is that most medical centers use gentamicin containing cement, rather than piperacillin or ceftazidime in bone cement, as their local antibiotic regimen and may produce a different outcome.

## Conclusions

The 10-year survivorship of implant with infection-free after two-stage revision THA was 82.2%, which was inferior to that of previous reports. Patients with obesity, liver cirrhosis, gram-negative organism and presence of sinus tract had a significant impact of the risk of reinfection.

## Abbreviations

BMI: Body mass index
CIs: Confidence intervals
CRP: C-reactive protein
*E. coli*: *Escherichia coli*
ESBL: Extended-spectrum β-lactamases
ESR: Erythrocyte sedimentation rate
GN: Gram-negative
HIV: Human immunodeficiency virus
HR: Hazard ratios
MRSA: Methicillin-resistant *Staphylococcus aureus*
MSSA: Methicillin-sensitive *Staphylococcus aureus*
PJI: Periprosthetic joint infection
SD: Standard deviation
THA: Total hip arthroplasty
